# The Refraction and Accommodation of the Eye

**Published:** 1886-09

**Authors:** 


*The Refraction and Accommodation of the Eye.* By E. LANDOLT, M.D. Translated by C. M. CULVER, M.A., M.D. Edinburgh: YOUNG J. PENTLAND. 1886.

The apparent simplicity of the adaption of lenses to imperfect vision is seen in the fact that the public consider watchmakers competent to help them in a selection of spectacles, or else they seek the aid of an ophthalmic optician, who, ignorant alike of physical optics and the physiology of the eye, advertises his ability to "suit all sights."

Undoubtedly the most important agent in the whole range of ocular therapeutics is the application of spectacles. The majority of patients who come for advice about their eyes require them, and without their aid many of the surgical procedures—such as strabismus operation—are sadly incomplete. At the same time, by help of competent knowledge, this form of treatment can be conducted on the sure basis of mathematical deduction: "in the doctrine of the anomalies of refraction and accommodation the connection between science and practice is more closely drawn together than in any part of medicine."

For twenty-one years, the classical work of Donders, published by the Sydenham Society, has been the only book for reference on this subject in the English language. It will always remain the great pioneer in this class of work, and is never likely to be entirely replaced. Having reached its majority, however, no wonder if a younger competitor should appear, in which the old work might be better arranged, whilst later scientific advances and the most recent methods of dealing with this branch of ophthalmic science might be introduced. Landolt, by his intimate knowledge of physical optics, by his reputation as a clinical surgeon, and by his past work and writings, seemed most competent to produce such a book; and it is interesting to note how largely we are indebted to the school at Utrecht (where our author was formerly a pupil of Snellen's) for advance in this direction.

The book before us is thoroughly up to date, and it is comprehensive. The clinical portion may be read alone, or studied after the theories of the subject have been considered. It contains also just what is wanted of physical optics.

Its form is admirable. It commences with the refraction of light, which is dealt with in the use of not very difficult mathematical problems. Lenses and spectacles—which are infinitely thin lenses—are fully described, and the advantages of the metric system—"Meterlinse," "Dioptry"—are well explained. The dioptric system of the eye is optically considered as the combination of three spherical refractive surfaces, with its principal and nodal points and conjugate foci. The first hundred pages of the book are devoted to this mathematical treatment of physical optics. It requires no high knowledge for understanding; but, if too technical, may be entirely omitted. The remaining five hundred pages are equally divided into a theoretical and a clinical portion. The theoretical portion deals with the refraction and the accommodation of the eye, and with the methods for their determination.

In considering the eye at rest—"static refraction"—the importance of the cornea (by separating the air and the aqueous) in deviating the luminous rays is insisted on; angle Alpha and angle Gamma are explained; and Helmholtz' dimensions of the dioptric system are given.

Emmetropia and ametropia in their various forms are discussed as "The Dioptric system of the Eye in its relation to the Retina." The methods for determining the refraction of the eye at rest are divided into subjective and objective: the former based upon the acuteness of vision, as tested by the use of reading-types and lenses in the ordinary way, or by some modification of this; or else by the aid of some optometer, examples of which are described and their working explained.

The objective determination of refraction is by means of the ophthalmoscope: (1) by the erect image; (2) by the inverted image; (3) by koroscopy.

The writer's observation in foreign clinics would incline him to believe that the objective method is much less used than in this country. Here the shadow test may be looked upon as the mainstay of practical refraction. It is in constant use in every clinic, and the results obtained by it are satisfactory both in accuracy and in saving of time. Landolt gives a good description of the method, and refers to Parent's writings on the subject; but he does not seem to work by it himself.

Under "Dynamic Refraction of the Eye," the parts concerned in accommodation—the ciliary body and the neighbouring structures—are fully described, together with their varying relations during change of focus; and an account is given of the various experiments upon which our present full knowledge of this subject depends.

The consideration of the amplitude of convergence and of accommodation is handled in a masterly way. The author's ophthalmo-dynamometer, and other methods of determination, are described.

Forty pages of the book are allotted to a separate chapter on astigmatism.

The part of the book of most interest to the surgeon is the clinical portion. It is, like the rest, well-written and scientific; but it is also eminently practical, and shows a common-sense consideration for the individuality of the patient, outside the mathematical formula for his dioptric derangement.

"There are many ametropes that do not need spectacles." "It is of the greatest importance to take into account the habits and fancies of the patient, and especially the glasses he has hitherto worn." "It is only actual experiment that can confirm the deliberations of the physician."

Strabismus, as being usually due to error of the dioptric system, is fully dealt with; and an interesting case is quoted from Horner of Zurich, showing the value of proper focussing even upon a detached retina.

Typical myopia is looked upon as the result of natural development under civilization: the arts require ocular work at a short distance, and only a short range of vision is necessary for intellectual progress.

Once given the elongation of the eye, which is the almost constant cause of this refractive anomaly, its posterior axis, where it is left unsupported either by Tenon's capsule or by the recti muscles, will more and more suffer under the influence of pressure; while the neighbourhood of the macula, where function is most elaborate, shows the greatest liability to morbid change. Thus may result the tissue-degeneration which constitutes a malignant myopia.

It is imperative upon all branches of our profession to lessen this growing tendency in over-taught children; and Landolt very fully discusses prophylaxis as well as treatment.

Spasm and paresis of accommodation, with the drugs that cause or cure them, bring the volume to a close.

The translation is excellent, and the letterpress all that can be desired.

